# Assessing environmental impacts and ecosystem services of Hops crop in Galicia, NW Spain: critical contributors for sustainable cultivation strategies

**DOI:** 10.1007/s11356-026-37623-0

**Published:** 2026-03-17

**Authors:** Adrián Agraso-Otero, Javier J. Cancela, María Fandiño, Ricardo Rebolledo-Leiva, Sara González-García

**Affiliations:** 1 CRETUS, Department of Chemical Engineering, School of Engineering, University of Santiago de Compostela, 15782 Santiago de Compostela, Spain; 2 GI-1716, Proyectos y Planificación, Departamento Ingeniería Agroforestal, Escola Politécnica Superior de Enxeñaría, Universidade de Santiago de Compostela, 27002 Lugo, Spain; 3 Department of Computing and Industries, Faculty of Engineering Sciences, Universidad Católica del Maule, Av. San Miguel, 3605 Talca, Chile

**Keywords:** Agriculture, Brewing Industry, Life Cycle Assessment, Pollination, Soil Erosion, Sustainability

## Abstract

Hops (Humulus lupulus) is a crop of great interest due to its use in the brewing industry; however, the environmental impacts associated with its cultivation remain largely unexplored. Therefore, this study provides the first evaluation of the environmental performance of hops cultivation using the Life Cycle Assessment methodology, with a case study of Galicia, NW Spain. The system boundaries, following a cradle-to-farm-gate approach, included all field operations throughout its entire lifespan (20 years), with primary data obtained directly from farmers. Impact categories such as Global Warming, Eutrophication, Ecotoxicity and Water Scarcity were considered. The main environmental hotspots identified were irrigation, due to the high crop water requirements, followed by agrochemical production and the associated on-field emissions. The results showed impacts of 3.05 kg CO₂ eq in global warming, 35.35 g SO₂ eq in terrestrial acidification or 70.3 m^3^ in water scarcity per kilogram of dry hop cones. Agricultural activities exert pressure on our natural ecosystems in different ways, for example, globally through the propagation of emissions or locally through the degradation of species-rich landscapes, with plants being the most affected taxon in this case. Moreover, two ecosystem services were assessed: pollination and soil erosion. Pollinator presence was found to be constant throughout spring and summer, with a ratio close to 50% of the regional maximum, while the effects of soil erosion control were estimated at €838 over the 20-year lifespan of the crop. With these results, the environmental performance of hop cultivation can be significantly improved, paving the way for more sustainable products within the brewing industry.

**Supplementary Information:**

The online version contains supplementary material available at 10.1007/s11356-026-37623-0.

## Introduction

Agriculture is a fundamental sector to the development and well-being of society. However, factors such as the low productivity and the ever-increasing global population density limits the progress of this sector (Fan et al. 2022). These constraints have led to the rise of intensive farming practices, characterised by high consumption of fertilisers and pesticides aimed at boosting crop yields, which in turn have resulted in significant negative environmental impacts, including soil degradation, biodiversity loss and increased greenhouse gas (GHG) emissions (Lago-Olveira et al. 2023; Tilman et al. 2006). Indeed, the importance of this issue is underscored by the fact that agriculture, forestry and land use are behind around 22% of total GHG emissions according to the Sixth Assessment Report (AR6) of the Intergovernmental Panel on Climate Change report (IPCC 2023).

Ecosystem services (ES), defined as “the contribution of ecosystems to the benefits that are then utilized in human activities”, play a crucial role in fostering sustainable agricultural practices, which in turn can lead to improved production yields and quality (FAO 2008). These ES encompass vital services such as pollination, carbon sequestration, water purification, soil erosion control and pest management (Lago-Olveira et al. 2025). Although LCA studies that integrate ES are still a minority, their importance is increasingly recognised as they provide a more comprehensive understanding of the environmental, social and economic impacts of human activities (Taelman et al. 2024). The examination of soil erosion is particularly important in assessing crop productivity, as it provides insights into the long-term fertility of the land and the potential nutrient losses resulting from erosion (Eurostat 2020). Additionally, biodiversity loss is a key factor in understanding ES such as pest control and pollination and their conservation could help create synergies that enhance both agricultural productivity and environmental sustainability (ICDA Sustainability 2021).

Hops (*Humulus lupulus*) is a perennial species with an expected productive life of at least 20 years (Afonso et al. 2020). Native to the temperate climates of the Northern Hemisphere, it is a climbing, herbaceous plant that produces inflorescences (Neve 1991). Female plants are those with flowers, which, when mature, are called cones. At the base of the cones are lupulin glands which produce compounds such as resins (alpha and beta acids) and essential oils, substances of great importance for the food (e.g. beer production) or pharmaceutical industries (Abram et al. 2015; Almaguer et al. 2014; Bocquet et al. 2018; Rettberg et al. 2018).

Global annual hop production was around 160,000 tonnes in 2022, with the world’s largest producers being the USA, Ethiopia and Germany (FAOSTAT 2022). However, in the case of the African country, there is some controversy, as it produces *Rhamnus prinoides*, not *Humulus lupulus*, which is similarly used for the brewing of a type of Ethiopian beer called ’tella’ (Jastrombek et al. 2022). Official statistics from the International Hop Growers’ Convention (IHGC) show hop production of about 118,457 tonnes in 2023, taking into account the official data from countries which are involved in the hop production focused on the brewing industry (IHGC 2024). In Spain, approximately 905 tonnes of hop cones were harvested in 2023, with more than 90% of national production coming from the province of León; while Galicia produced only 15.7 tonnes, despite having a significant brewing industry (Ministry of Agriculture, Fisheries and Food 2023).

The best-known use of hops is for brewing beer, which is the world's oldest biotechnological process, with evidence of brewing dating back 8,000 years to the Middle East (Korpelainen and Pietiläinen 2021). This beverage holds great significance in Spain, as it is included in the Mediterranean diet, which is recognised as UNESCO Intangible Cultural Heritage (Bach-Faig et al. 2011). Although Spain is not a leading hop producer in Europe, lagging behind countries such as Germany, the Czech Republic, Poland and Slovenia, it ranks second in beer production within the European Union behind Germany, with 41.5 and 87.7 million hectolitres produced in 2023, respectively (Brewers of Spain 2023). Moreover, Hijos de Rivera, based in Galicia, is the fourth-largest beer producer in Spain (third if only Spanish-owned companies are considered) and enjoys significant international prestige, with some of its products recognised among the best in the world (Brewers of Spain 2023; World Beer Awards 2024). Promoting and incentivising local hop cultivation in the region is therefore a key pillar of sustainability and environmental policies, with only 1 g of hops estimated to be required to produce one litre of beer (Alfonso 2019). Another of the most important uses of this plant is within the field of traditional medicine, as its properties make it a good remedy to combat anxiety, coughs, spasms, fever or inflammation, as it has antioxidant, anti-inflammatory, anticancer and antimicrobial activity (Alonso-Esteban et al. 2019; Chattopadhyay 2010; Zanoli and Zavatti 2008).

Plant production capacity is typically between 30 and 70% of the theoretical maximum, with yields varying from year to year (Donner et al. 2020) these variations are higher in the last years due to climate change effects with extreme events such as drought or heat waves (Potopová et al. 2021) or natural disasters (e.g., the 2024 river floods). To maximise these yields, it is essential to choose a suitable location with good soil conditions (Cancela et al. 2022) and water availability, as well as good management of the necessary fertilisation and agrochemicals (Biendl et al. 2015; Donner et al. 2020). This is where certain areas of Galicia stand out thanks to their unique characteristics for hop cultivation, as the crop requires a climate with frequent rainfall to develop its characteristic bitterness, as well as adequate sunlight to enhance its aromatic profile (Lutega 2024). Irrigation is a key factor, particularly in regions with moderate annual rainfall or during the summer period, as it is a sensitive time because it is when flowering and cone formation occur, as well as the production of alpha and beta acids (De Keukeleire et al. 2007; Fandiño et al. 2015; Guimarães et al. 2021).

In the agri-food sector, the environmental implications of agricultural practices are commonly evaluated using the Life Cycle Assessment (LCA) methodology, which enables a comprehensive assessment across multiple impact categories (Borz et al. 2025; Giffard et al. 2022). However, in the context of beer production, the environmental impacts associated with hop cultivation are often omitted or substantially simplified. This may be due to several factors: i) hops are frequently considered negligible contributors to the overall environmental footprint of beer (Saget et al. 2022); ii) hop cultivation is excluded from system boundaries (Ooyama et al. 2025); or iii) proxy inventories from other crops, such as barley, are used instead (Amienyo and Azapagic 2016). As a consequence, comprehensive environmental assessments specifically addressing the agricultural phase of hop cultivation remain scarce.

In this context, this study evaluates the potential environmental impacts of hop cultivation in Galicia (north-western Spain), a region characterised by favourable agroclimatic conditions for hop growing. Despite the current reduced hop cultivation area in Galicia, historical evidence indicates that the region was a key hop-producing area during the mid-twentieth century, reaching up to 236 ha in the 1960 s, with a demonstrated potential for producing high-quality hops (Breuer 1980; Fandiño & Cancela 2020). The subsequent decline in hop surface was primarily driven by changes in Spanish agricultural policies rather than by agronomic or climatic limitations. Moreover, recent studies suggest that Galicia’s climatic conditions may be better adapted to future climate change scenarios than those of other Spanish hop-growing regions, such as León, due to a lower frequency of heat waves and a reduced dependence on irrigation (Potopová et al. 2021). Consequently, the objective of this study is to identify the main environmental hotspots of hop cultivation and to propose potential strategies to improve its environmental performance using the LCA methodology, explicitly incorporating ecosystem service–related aspects such as biodiversity loss and soil erosion to provide a more holistic assessment of hop-growing systems (Costa et al. 2020; Martínez-García et al. 2025).

## Materials and methods

### Study area and evaluated scenario

**Fig. 1:**
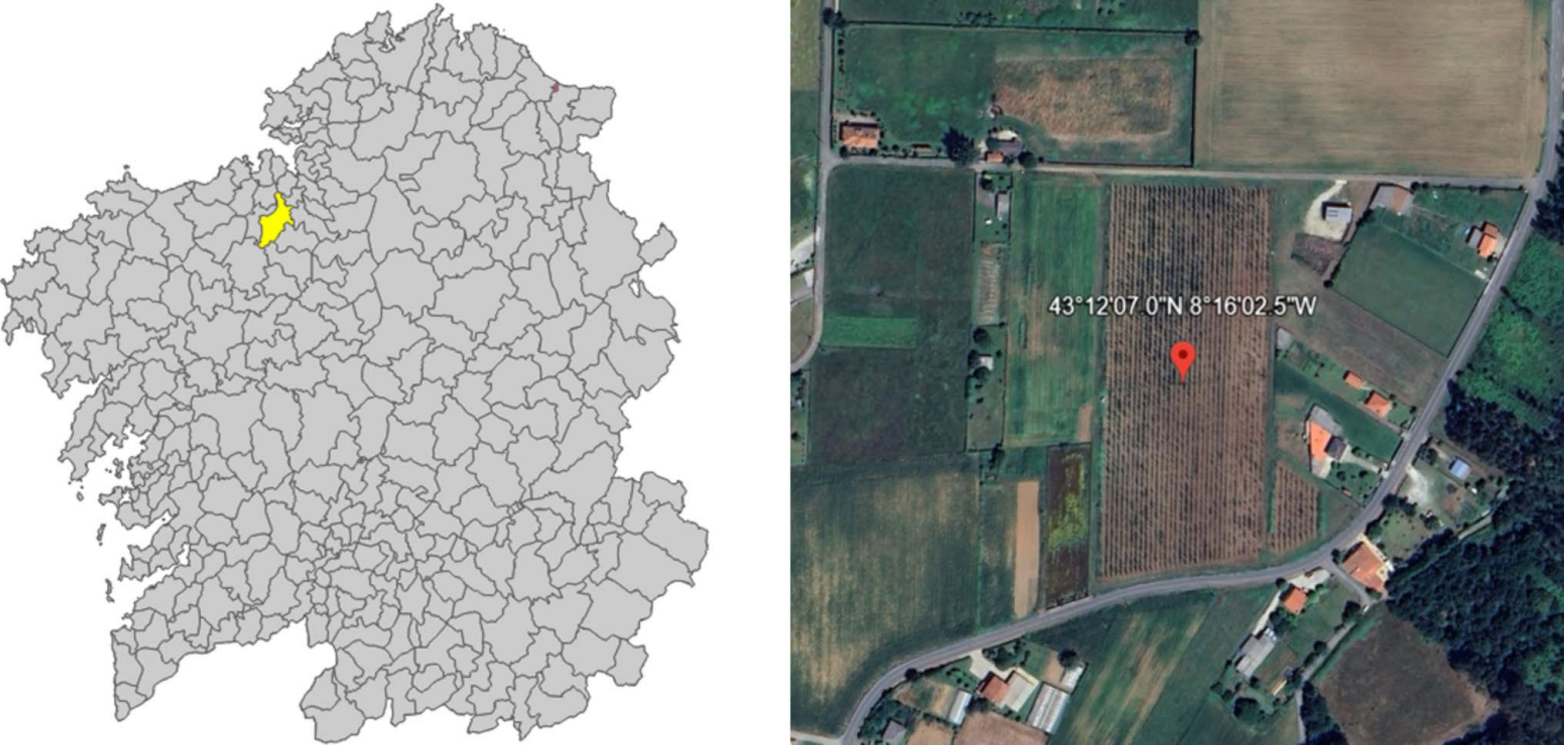
Map of the Galician region with the council of Abegondo highlighted in yellow, showing the location of the hop field, alongside a picture of the field

This study was carried out in collaboration with LU.TE.GA., a hop farm production cooperative in Galicia, in a 2-hectare farm in ‘Presedo’ (Abegondo), A Coruña province, northwestern Spain (43° 12′ 7.0’’ N, 8° 16′ 2.5″ W, elevation 165 m) (Fig. 1). The region is characterised by constant rainfall (928.3 mm/year) and moderate temperatures throughout the year (Global Modeling and Assimilation Office (GMAO) 2015). The studied farm is under a conventional growing system, with an annual irrigation between 70 to 110 mm, applied from May to September. The plant variety grown is *Nugget*, with an expected lifespan of 20 years and a maximum production of 2.2 tonnes per year (Lutega 2024), more details about the crop system are available in Cancela et al. (2022). Additionally, the reported data are considered representative of the agricultural practices required for hop cultivation.

### Environmental assessment

The environmental impacts of this system will be quantified and analysed using an attributional LCA methodology, in accordance with the guidelines established in ISO 14040 and ISO 14044 (ISO 2006a, b). The following sections outline the different phases of the methodology: goal and scope definition, life cycle inventory analysis, life cycle impact assessment and interpretation of the results.

#### Goal and scope definition

The goal of the manuscript is to determine the environmental impacts of a conventional hop cultivation system in Galicia. Moreover, this will help identify the critical stages of the crop system to target future improvements aimed at achieving the most environmentally sustainable production possible. The functional unit (FU) serves as the reference unit for calculating all inputs and outputs within the system under study, and it was established as 1 kg of dry hop cones (10% moisture) to account for the different yields throughout the plant’s life cycle and minimise the production-related impacts (Nemecek et al. 2015).

**Fig. 2:**
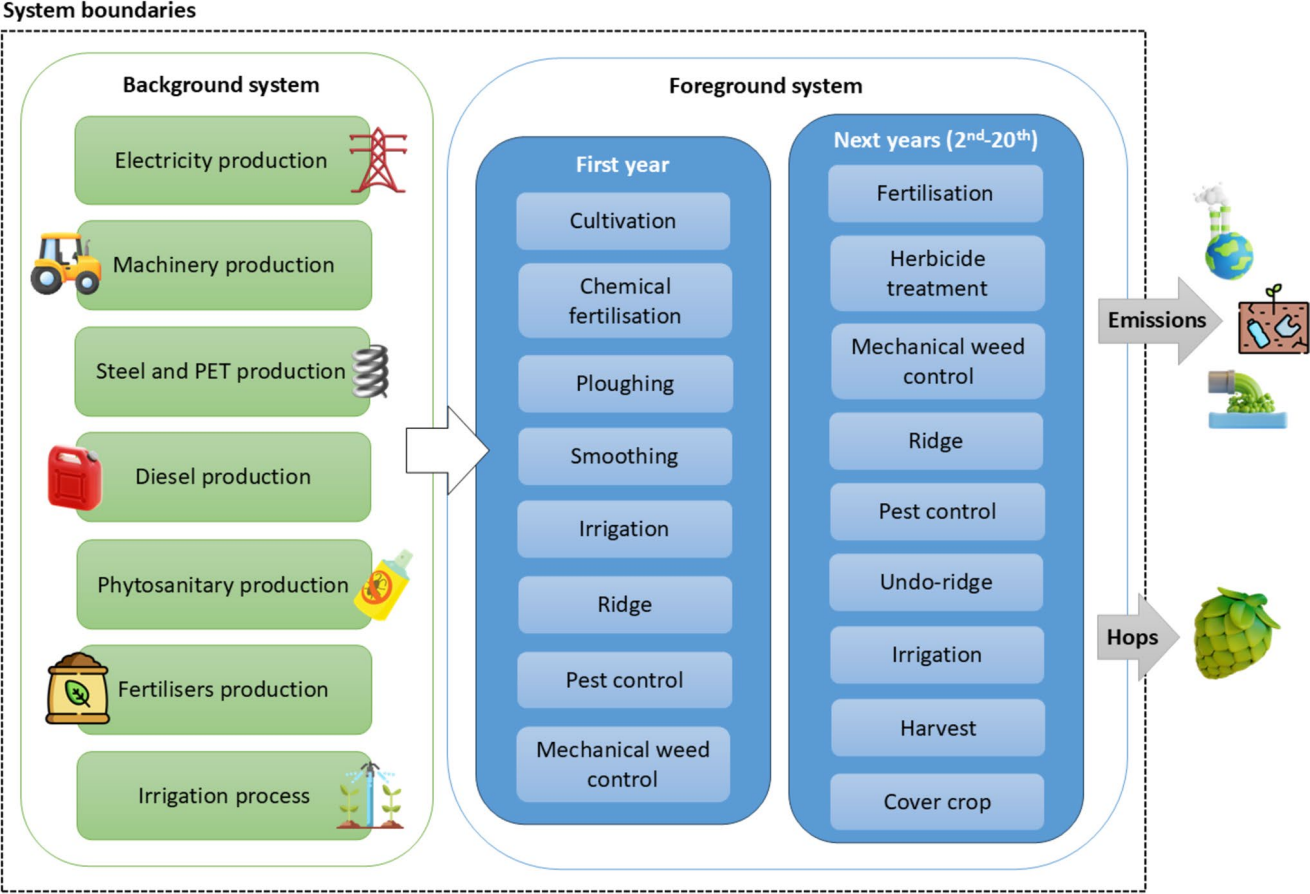
System boundaries of hops cultivation with the main activities and processes considered

A cradle-to-farm-gate approach was followed (Fig. 2), with the system boundaries including raw material extraction (e.g., fossil fuels, steel), agrochemicals production, machinery elaboration and all field operations carried out. Although cone drying is not performed within the system boundaries, as it is not carried out at the farm, the impacts are expressed per kilogram of dry hop cones to ensure consistency with market specifications. No allocation is performed, as the total environmental impacts are associated with the only system's product, the hop cones, as residues from the plants are shredded and deposited into the soil, acting as an organic amendment. Additionally, the lifespan of the crop is 20 years as explained above, capturing production for each year and the various operations carried out in the planting year as well as in the subsequent years, during which production gradually increases, reaching its maximum from the fourth year onwards.

##### System under study

The operations and requirements of the hops are different in the first year than in the following ones. In the planting year, which is the initial year, the hops are not harvested but grown and maintained. Planting is carried out using rhizomes, followed by fertilisation and soil preparation between March and May. From June to August, daily irrigation is implemented to support optimal crop establishment. Concurrently, pest management treatments are conducted from March to August to control infestations of pests such as mildew or green caterpillars. By the end of the first year, mechanical weed control measures are applied to ensure proper soil maintenance (Lutega 2024).

In the following years, the hop cones are harvested, increasing their yield from 30 to 70% in the first and second years, respectively, and then to 100% from the third to the twentieth year, which is the end of the lifetime of the system. Over the years, agricultural practices have remained largely unchanged. These practices typically commence with nitrogen-based chemical fertilisation to ensure an optimal nutrient composition that supports plant growth, reaching a height of 6 m at the end of June. Specifically, this involves the application of 900 kg/ha of NPK fertiliser, 250 kg/ha of calcium ammonium nitrate (NAC27) and 200 kg/ha of ammonium nitrate 34.5%. In addition, a series of herbicide and pest control treatments are applied from March until the harvest in September to prevent crop damage. To protect the plant, ‘aporcados’ are made, i.e. soil is accumulated in small mounds around the crop line. Weed control is also applied, and one month before harvest, cover crops are sown between the rows to protect the plants and avoid soil erosion (Biendl et al. 2015), a practise carried out on a year-to-year basis (Lutega 2024). The full data of the treatments realised can be seen in Tables S1-S3 in the Supplementary Material (SM).

The irrigation is therefore carried out over the entire lifespan of the plant for 72 days each season and with an average of 4 h of use per day, with drip irrigation by an electric submerged pump. In other words, to the whole hop farm (2-ha), a total of 24,000 L of water are used per day, resulting in a high-water demand for this crop, alongside an overall electricity demand of 540 kWh. The necessary infrastructure includes 218 wooden posts, 4,500 m of steel wire and 28,600 m of rope. Regarding the organic residues generated during harvesting, each plant produced 52 kg of biomass residues, amounting to a total of 114.4 tonnes.

#### Life cycle inventory

The environmental impacts were calculated using a combination of primary data, obtained directly from the farmers by sending out a series of questionnaires concerning their hop growing practices (e.g. fuel consumption or amount of agrochemicals applied). Secondary data were used to model the processes that constitute the background system, i.e. those that are not under the control of the farmers (e.g. such as fertiliser, fungicide or fuel production) and are extracted from the Ecoinvent® database version 3.8 (Ecoinvent 2024). The origin of each data and the inventory extracted from the Ecoinvent® database are provided in Tables S5–S6 in the SM. The inventory data of the hop production used are shown in Table 1.

**Table 1:** Inventory data associated with the production of hops (per hectare of land)

**Inputs**	**Value (first year)**	**Value (next years)**	**Unit**
Tractor	13.3	40.5	kg
Diesel	790	1250	kg
Tillage	47.6	58.4	kg
Water	869	869	m^3^
Electricity	540	540	kWh
N (inorganic fertiliser)	141	141	kg
P_2_O_5_ (inorganic fertiliser)	135	135	kg
K_2_O (inorganic fertiliser)	135	135	kg
NAC27	250	250	kg
Ammonium Nitrate	200	200	kg
Pesticide	12.1	12.1	kg
Steel	7.87	7.87	kg
Polypropylene	14.4	14.4	kg
Softwood posts	1.02	1.02	m^3^
**Outputs**	**Value (first year)**	**Value (next years)**	**Unit**
Product			
Hop cones	39.6^*^		t
Emissions to air			
Carbon dioxide (iLUC)	48	48	kg
Carbon dioxide (dLUC)	−2.89	−2.89	t
Dinitrogen monoxide	4.65	4.65	kg
Nitrogen dioxide	8.34	8.34	kg
Ammonia	14.4	14.4	kg
Dimethylurea	0.04	0.04	kg
Sulphur	0.36	0.36	kg
Copper sulfate	0.08	0.08	kg
Trifloxystrobin	0.01	0.01	g
Penflufen	0.01	0.01	kg
Emissions to water			
Phosphate (groundwater)	0.21	0.21	kg
Phosphate (river)	0.72	0.72	kg
Nitrate	461	461	kg
Dimethylurea	4.68	4.68	g
Sulphur	0.04	0.04	kg
Copper sulfate	9.00	9.00	g
Trifloxystrobin	1	1	mg
Penflufen	1.28	1.28	g
Emissions to soil			
Dimethylurea	0.42	0.42	kg
Sulphur	3.60	3.60	kg
Copper sulfate	0.81	0.81	kg
Trifloxystrobin	0.15	0.15	g
Penflufen	0.12	0.12	kg

*Total amount of dry hop cones (10% moisture) in the entire lifespan

Several empirical models are employed to estimate field emissions resulting from the application of pesticides and fertilisers: the Intergovernmental Panel on Climate Change (IPCC) Guidelines for National Greenhouse Gas Inventories, which are used to quantify direct and indirect N_2_O emissions (IPCC 2019); the European Environment Agency (EEA) and the European Monitoring and Evaluation Programme (EMEP) (*EMEP/EEA* 2023) for the emissions of nitrogen dioxide (NO_2_) and ammonia (NH_3_); the model by Faist Emmenegger et al. (2009) for the calculation of nitrate (NO_3_^−^) emissions, assuming a clay content of 5.25% and an average effective root depth of 2 m. In addition, local conditions, including rainfall patterns (928.3 mm/year) and soil carbon content (81 t C·ha^−1^), were considered to estimate nitrate mobility in the soil and, consequently, its potential contribution to marine eutrophication. Additionally, the SALCA-P method, carried out by Agroscope (Prasuhn 2006), was used for phosphorus emissions through leaching and run-off; and the European Commission's Product Environmental Footprint Category Rules (European Commission 2018) to estimate the emissions of active ingredients associated with the use of different phytosanitary products into air, water and soil.

As far as field emissions from land use changes are concerned, this study considered indirect land use change (iLUC) emissions, which are defined as those emissions due to the land use transformation as a consequence of land occupation to be studied for hop cultivation. Similarly, direct land use change (dLUC) emissions, i.e., emissions related to the management of the green residues from the plants that are shredded and deposited into the soil (Schmidt et al. 2015) were also considered. To quantify iLUC, the method of Schmidt et al. (2015) was used, where data on the amount and time of land occupation as well as the productivity provided by Haberl et al. (2007) were given. Finally, to arrive at CO_2_-equivalent emissions, a specific factor of 0.042 t CO2 per hectare and year, considering the attributional approach, was established for arable land. Details of the calculation of iLUC are provided in Table S7 of the Supplementary Materials. Regarding dLUC emissions, a value of 16% was assumed for the organic carbon remaining in the soil (Fang et al. 2019), and the carbon content was estimated based on the characterisation of hop pruning residues reported by (Rubira et al. 2024).

An uncertainty analysis was carried out on the inputs obtained from the Ecoinvent® database to reinforce the results. The Monte Carlo simulation was performed in SimaPro® software with 2,000 iterations and a 95% confidence interval to propagate uncertainties and assess their impact on the final results, which are presented in Table S8 in the Supplementary Material.

#### Impact assessment

The environmental impact of the farm was assessed on the basis of the following impact categories with high importance in the agricultural sector: Global Warming (GW), Stratospheric Ozone Depletion (SOD), Terrestrial Acidification (TA), Freshwater Eutrophication (FE), Marine Eutrophication (ME), Terrestrial Ecotoxicity (TET), Freshwater Ecotoxicity (FET), Marine Ecotoxicity (MET). These impact categories were quantified using the ReCiPe 2016 v1.07 Hierarchist Midpoint World (2010) method (Huijbregts et al. 2017). Additionally, Water Scarcity (WS) was assessed using the AWARE method v1.2c (Boulay et al. 2017), which accounts for both water consumption and regional water availability by applying a regional water scarcity factor that reflects local water demand and supply. Since the geographical location of hop cultivation did not change, the regional factor was consistently applied across years. All these indicators were evaluated with SimaPro® software v10.0 (PRé Sustainability 2022). For the biodiversity assessment, the Potential Species Loss (PSLglo) was quantified based on the methodology developed by Chaudhary et al. (2015). Additionally, soil erosion and pollination were estimated using the models described below.

##### Pollination

Pollination analysis was carried out using free Integrated Valuation of Ecosystem Services and Tradeoffs (InVEST®) v3.14.3 crop pollination model (Natural Capital Project 2024a). To assess spatial and temporal variations, QGIS software v3.34.12 was used (QGIS Development Team 2024). The InVEST® crop pollination model focuses on wild bees as a key pollinator and on two main aspects: pollinator supply and pollinator visits to crops (Natural Capital Project 2024a). The model converts land cover into an index of suitability (0–1) for bees, producing a pollinator source map where higher scores indicate greater relative bee abundance. Land use/land cover (LULC) map was downloaded from CORINE Land Cover for 2018 using the Copernicus Land Monitoring Service (European Environment Agency 2018). Bees’ species abundance in Spain was sourced from Bartomeus et al. (2022), based on the Iberian bees database, while other parameters for the guild and pollinator table were derived from varied literature sources (Natural Capital Project 2022; Sanguet et al. 2023; Zhao et al. 2023). The land use preceding hop cultivation was classified as natural grassland.

##### Soil erosion

Soil erosion was modelled following the Sediment Delivery Ratio model (SDR) of INVEST® v3.14.3 (Natural Capital Project 2024b), with QGIS software v3.34.12 employed for map interpretation and modification (QGIS Development Team 2024). This model enables the quantification and mapping of sediment movement caused by factors such as streamflow, slope or external factors like wind or rainfall. It helps identify areas where sediment may accumulate, which is crucial for designing strategies focused on their proper management. This not only helps mitigate runoff phenomena but also contributes to maintaining agricultural productivity.

The sources of all the inputs required for the simulation of this model are provided in Table S4 in the SM. For the biophysical table, a support practice factor (P-factor) specific to Spain, as provided by Panagos et al. (2015), was used for all land use classes (LUC). Meanwhile, the cover management factor (C-factor) was adopted from the study by Marques et al. (2021), as the same LUC were used. As a result, the program generates a series of maps quantifying parameters such as potential soil loss, expressed in tonnes per pixel per year, based on the USLE equation (Eq. 1):

1 $${usle}_{i}={R}_{i}\cdot {K}_{i}\cdot {LS}_{i}\cdot {C}_{i}\cdot {P}_{i}$$

where:

- R_i_: rainfall erosivity (MJ⋅mm/(ha⋅hr⋅yr)),
- K_i_: soil erodibility (t⋅ha⋅hr/(MJ⋅ha⋅mm)),
- LS_i_: slope length–gradient factor (unitless),
- C_i_: cover-management factor (unitless),
- P_i_: support practice factor (unitless).

The detailed methodology is reported in the paper by Lago-Olveira et al. (2025).

## Results

### Environmental impact profile

The results presented in Table 2 show the environmental impacts of a hop plantation over its entire lifespan. Expressing the results while considering production yield provides a balanced perspective between land productivity and the environmental impact of the agricultural operations carried out. This production-focused approach is also crucial for incorporating the cultivation impacts into later processing stages, such as beer production.

**Table 2:** Environmental impacts per 1 kg of dry hop cone (10% moisture)

Impact category	Unit	Value
GW	kg CO_2_ eq	2.96
SOD	mg CFC_11_ eq	29.8
TA	g SO_2_ eq	34.9
FE	g P eq	1.09
ME	g N eq	15.7
TET	kg 1,4-DCB	14.7
FET	g 1,4-DCB	137
MET	g 1,4-DCB	180
WS	m^3^	35.6
PSLglo	PDF·year	1.75·10^–15^

GW: Global Warming, SOD: Stratospheric Ozone Depletion, TA: Terrestrial Acidification, FE: Freshwater Eutrophication, ME: Marine Eutrophication, TET: Terrestrial Ecotoxicity, FET: Freshwater Ecotoxicity, MET: Marine Ecotoxicity, WS: Water Scarcity, PSLglo: Potential Species Loss

Currently, there are no published studies focusing on the environmental impacts of the agricultural phase of hop production, with research only addressing it as part of the beer supply chain. The only related information found was a study by Bristol (2022), in which the environmental impacts of American hops in the beer supply chain were estimated. These authors considered only two impact categories: GW and ME, with total impacts for the agricultural stage of 2.08 kg CO2 eq and 4.46 g N eq per kg of hop pellet, respectively. Emissions related to fertilisers and the irrigation process account for 84% for GW and 64% for ME (Bristol 2022). The percentage of emissions associated with the irrigation system constitutes 30% of the agricultural phase impacts in GW, which is consistent with the results obtained in this study, changing from 28% in the first year to 22% in subsequent years. In contrast, the impacts related to the application of agrochemicals change from 48 to 38% of the GW category, which is slightly lower than those reported by Bristol (2022), as in that case, the application of pesticides and tillage were excluded from the system boundaries. Regarding ME, Bristol (2022) identified electricity and irrigation as the main contributors. However, this is not consistent with the findings of this study, where the main impacts in this category stem from the decomposition of nitrogen-based fertilisers into nitrate. These differences could be attributed to variations in fertiliser application rates, although Bristol (2022) does not provide specific data on this aspect.

**Fig. 3:**
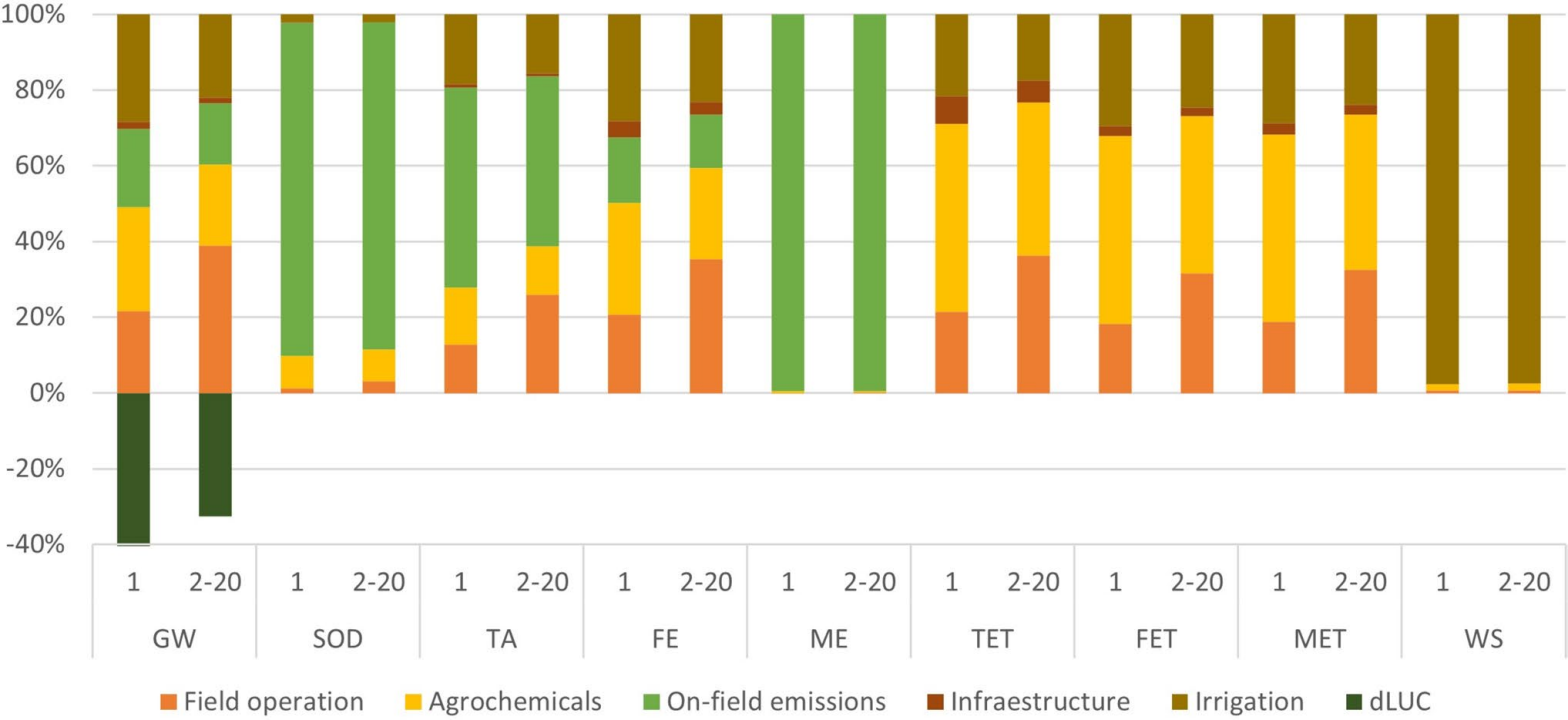
Percentage distribution of environmental impacts per year in each analysed category and their causes. GW: Global Warming, SOD: Stratospheric Ozone Depletion, TA: Terrestrial Acidification, FE: Freshwater Eutrophication, ME: Marine Eutrophication, TET: Terrestrial Ecotoxicity, FET: Freshwater Ecotoxicity, MET: Marine Ecotoxicity, WS: Water Scarcity

Figure 3 shows the distribution of environmental impacts across the analysed impact categories, distinguishing between those generated in the first year of cultivation and those from subsequent years. It is important to highlight that the main difference between the planting year and the production years is the increasing contribution of field operations to most impact categories, except for SOD, ME and WS where on-field emissions and irrigation are the main contributors. The rationale behind these results is the increased intensity of field operations, which leads to higher consumption of machinery and diesel, as well as increased emissions from fuel combustion.

#### Global warming

Impacts on GW are primarily driven by CO_2_ and N_2_O emissions. The former originates from the high diesel consumption associated with field operations such as ploughing, as well as from pumping and fertiliser production, while the latter is generated from the decomposition of nitrogen-based fertilisers. In the first year, GW impacts are evenly distributed among different sources, including field operations (i.e., machinery use and diesel combustion), agrochemicals (including their production and the water needed for their dispersion), on-field emissions (from agrochemical applications for soil nutrition and pest protection and emissions derived from indirect land use change) and diesel consumption for irrigation. Among these, diesel consumption for pump operation is the main driver, accounting for 28% of the total impacts, followed by agrochemical production at 27%. From the second year onwards, field operations become the dominant contributor, responsible for 39% of the impacts in this category due to the increased number of required activities, such as hop harvesting, and the CO_2_ generated from the combustion of the diesel required by the tractor. Among field operations, those related to soil preparation and management are responsible for most impacts, ranging from 62% in the first year to 80% in subsequent years. Notably, dLUC plays a crucial role in this category, offsetting up to 2.89 t CO₂ eq through the retention of biomass residues in the soil, more than twice the N₂O emissions resulting from fertilisation; therefore, it is represented with a negative value. This negative dLUC value reflects an increase in soil organic carbon. While the sequestered carbon is relatively stable under current management practices, uncertainties remain regarding its long-term retention (Slepetiene et al. 2025). These residues prevent CO₂ release into the atmosphere, counteracting up to 42% of the emissions generated in the first year, with the offset decreasing to 33% in subsequent years.

#### Stratospheric ozone depletion

In the case of SOD, dinitrogen monoxide emissions from fertiliser application are the primary contributors, with this substance accounting for almost all the total impacts. Additionally, over 86% of the impacts in the entire lifespan of the crop are attributed to on-field emissions, as all fertilisers used are nitrogen-based. This is followed by the impacts related to agrochemicals production, which contributes 8% of the total impacts. The importance of emissions derived from the use of nitrogen fertilisers for ozone depletion has been widely supported in the literature (Fajardo et al. 2025; van den Oever et al. 2024).

#### Terrestrial acidification

In TA, the impacts originate from NH₄⁺, NOₓ and SO₂, with the former accounting for 53% of the total loads. The main driver in this category is on-field emissions derived from nitrogen fertilisers use, representing 53% of the impact profile in the first year and decreasing to 45% in subsequent years, all driven by ammonia emissions. Field operations, irrigation and fertilisers production also have a significant contribution due to nitrogen oxides generated from diesel combustion, as well as ammonia and sulphur dioxide emissions from fertiliser production.

#### Freshwater and marine eutrophication

In FE, emissions are primarily driven by phosphates, followed by COD, due to water emmisions generated during fertilisation and those resulting from the construction of the irrigation infrastructure. Additionally, the combined contribution of irrigation and agrochemicals production is the most important in the first year, accounting for approximately 58% of total emissions, and decreases by 47% in subsequent years mainly due to the increasing weight of field operations. On the other hand, nitrate is the predominant, and almost sole, contributor to impacts in ME, with more than 99% of the total. This is primary due to the transformation of nitrogen present in fertilisers applied to the field, which subsequently leaches into water bodies, contributing to eutrophication. As a result, on-field emissions account for 99% of the total impacts in this category, highlighting the importance of optimise the amount used to mitigate its environmental impacts.

#### Terrestrial ecotoxicity, freshwater ecotoxicity and marine ecotoxicity

The impact profiles of TET, FET and MET are quite similar, with irrigation and agrochemical production identified as the main hotspots. This is primarily due to the emissions of substances such as copper and zinc, while for TET, nickel and chromium also have several contributions (12%). Among these substances, copper emissions are the main focus, accounting for 69% of the total in TET, 65% in FET and 59% in MET.

The Monte Carlo uncertainty analysis shows limited variability in impact categories such as GW, SOD, TA or ME, with low standard deviations relative to the mean values obtained, indicating that the conclusions are robust despite uncertainties in the input data. with low standard deviations relative to the mean values obtained. However, higher variability is observed in marine and freshwater ecotoxicity categories, reflecting the greater uncertainty associated with toxicity characterisation factors and the complexity of modelling pollutant effects in environmental compartments.

#### Water scarcity

As expected, the vast majority of impacts in this category are attributed to the irrigation process, which requires approximately 12 m^3^ per hectare per day. Although the irrigation volume is constant across years, the relative contribution of water use to water scarcity impact varies slightly due to changes in other field operations. Nevertheless, the overall water scarcity impact remains very similar across years, with only minor variations attributable to these differences in field activities. This becomes a problem, as in areas with limited water resources, competition between agriculture, human consumption and ecosystems increases, so the objective should be focused on how to reduce the water demand from the nearest watershed to alleviate the region’s water stress and ensure water availability. Since the amount of water required by the crop is difficult to reduce, one potential solution to mitigate this pressure could be the use of reclaimed water, as it can offer a sustainable alternative while reducing the reliance on freshwater sources (Wang et al. 2017). Moreover, irrigation water management tools could be used to apply hop water requirements more precisely. Specific studies at a local level are required to determine the irrigation requirements, which could reduce the extractions of subsurface water resources (Fandiño et al. 2015).

Once all these categories have been analysed, it can be concluded that further research is needed on how to make this sector as sustainable as possible, given that agriculture is essential for supporting human population growth, yet it is also responsible for a significant contribution to global warming emissions. Therefore, considering the hotspots identified in this study, the current irrigation system exhibits significant energy and diesel consumption, meaning that alternatives based solely on electricity or solar energy could help mitigate some of these impacts (García et al. 2018). Furthermore, a large amount of fertiliser is applied, leading to high field emissions, which could be partially reduced with reclaimed water, naturally supplying nutrients like nitrogen or phosphorus that the plants require for growth (Alcaide Zaragoza et al. 2022). Finally, field operations are also abundant, requiring substantial machinery and diesel consumption. Thus, implementing natural solutions, such as vegetation cover to reduce tillage operations or biological pest control to decrease pesticide application, could help alleviate these issues (Martínez-García et al. 2025). Other potential actions are relative to using drones or terrestrial robots to reduce tillage practices with tractors (Shan et al. 2024).

### Global potential species loss

**Fig. 4:**
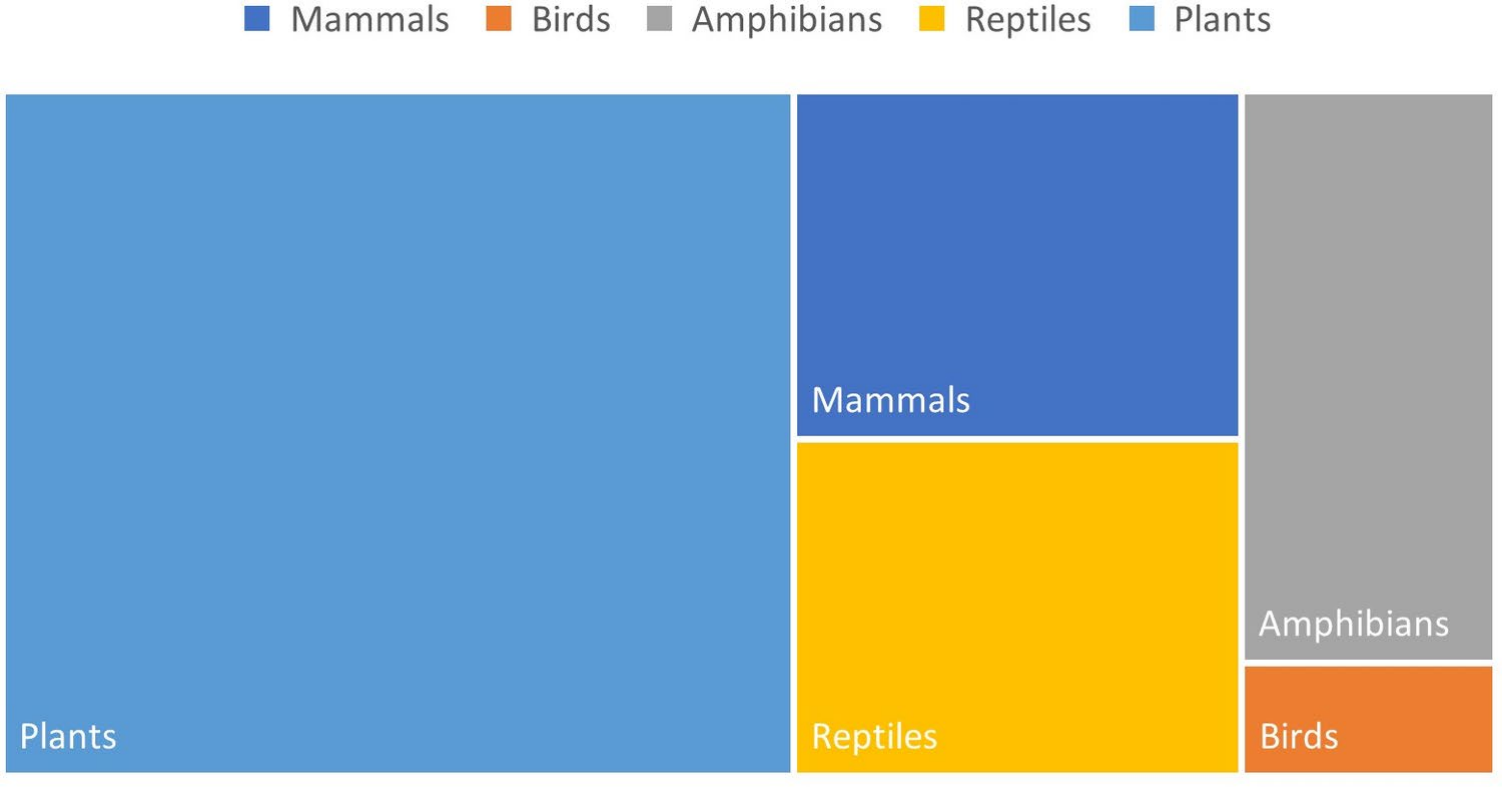
Distribution of impacts across each of the taxonomic groups studied

The global impact of potential species loss is established at 1.75·10^–15^ PDF·year per kilogram of hop cones harvested, or 3.46·10^–12^ PDF·year per hectare, 99% of which corresponds to infrastructure production processes, specifically to softwood production. These values are relatively low compared to other agricultural studies that include this parameter. For example, a result of 9.81·10⁻⁹ PDF·year is reported for old lands and 2.88·10⁻^1^⁰ PDF·year for new land in two-year crop rotations in Egypt per hectare of land (Lago-Olveira et al. 2023). Moreover, Lucas et al. (2021) study the impacts in different Brazilian ecoregions per kg of soybean produced, with values ranging from 1.12·10⁻^12^ to 2.89·10⁻^13^ PDF·year. Of these impacts, the majority are due to the annual occupation of the land for hop cultivation (78%), while only 22% is attributed to the transformation of land use from a natural state, such as pasture, to agricultural use. Considering the impacts on each of the studied taxa and the fact that the case study is located in the PA0406 ecoregion (Cantabrian mixed forest) (Olson et al. 2001), plants would be the most affected taxon in terms of biodiversity loss (53%) due to this crop cultivation, as they are not only the most abundant group in the ecoregion but also the least mobile. In contrast, birds would be the least affected due to their greater mobility (3%) (Fig. 4).

Therefore, although the biodiversity loss generated by this crop is not very high, it could be compensated through natural solutions that promote its presence, such as the implementation of vegetation cover or refuges for small invertebrates (Martínez-García et al. 2025). In this region, spontaneous vegetation grows in the inter-row during the whole season (Fandiño et al. 2015), and its usual seed is a mixture of turnip and pepper to use as a cover crop in the last month of the vegetative cycle of hops (Lutega 2024). This aspect decreases the pressure of PDF compared with other agronomic systems studied.

### Pollination

**Fig. 5:**
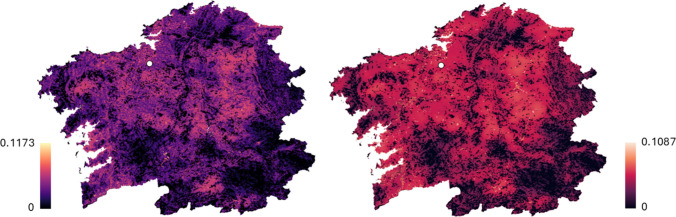
Pollinator abundance ratio in Galicia in spring (left) and summer (right). The white circle indicates the location of the hop cultivation orchard

It can be clearly seen (Fig. 5) how the abundance of pollinators in Galicia is different in summer than in spring because although the maximum abundance ratio is not very different, the distribution of bees in the territory does vary, obtaining specific ratios for the hop-growing area (represented as a white circle) of 0.0564 for spring and 0.0580 for summer. This represents 48% of the maximum value obtained in the Galician territory in spring, whereas in summer it increases to 53%. Since hops are harvested in September and considering that pollinator abundance is relatively consistent between spring and summer, it would be interesting to study how to reduce the amount of phytosanitary products used by shifting towards natural pest control strategies that focus on supporting pollinator populations. It’s relevant to mention that *Humulus lupulus* is a wind-pollinated species, where males are usually regarded as negative since male plants fertilise the females and avoid the formation of the valuable ‘hops cones’ (Small 2016). To prevent pollination, hop growers routinely remove male plants; therefore, the presence of wild bees is not detrimental to hop production (Neve 1991).

Although pollination does not directly affect yield, the pollination indicator is included here not as a crop-dependent service but as a proxy for habitat quality and ecological integrity. Wild bees are sensitive to landscape structure, pesticide use and vegetation diversity, and their potential presence reflects broader regulating and supporting ecosystem services in the hop-growing system (Goulson et al. 2015). Including this indicator allows us to capture ecological co-benefits of hop cultivation in Galicia that are not reflected in yield-based metrics and to contextualise trade-offs between production intensity and environmental performance. While these results represent a model-based approximation and more accurate assessments would require direct field observations of pollinator presence and abundance, they nonetheless highlight the role of hop plantations in supporting biodiversity-related ecosystem services.

### Soil erosion

**Fig. 6:**
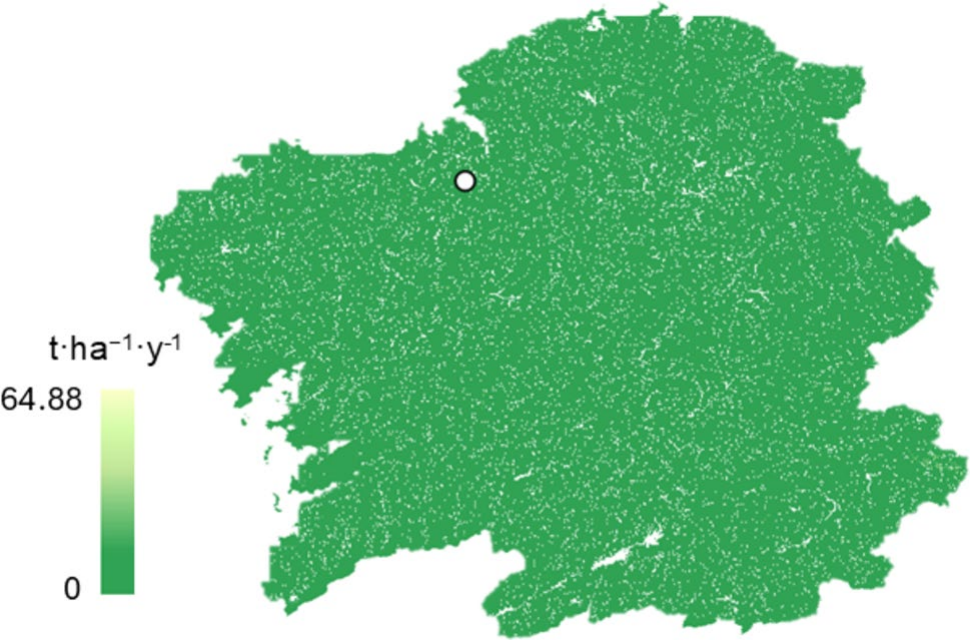
Soil erosion map according to USLE equation for Galician territory. The white circle indicates the location of the hop farm

The soil erosion for Galician territory according to USLE equation can be seen in Fig. 6, with the pilot represented with a white circle. In most of the territory, soil losses are generally close to zero across the entire region, with a specific value of 0.47 tonnes per hectare and year for the pilot area. According to the classification of soil erosion intensity (Eurostat 2025), the erosion level in the evaluated scenario is considered low, as it is below 5 tonnes per hectare per year.

To validate the obtained results, Van Rompaey et al. (2003) analysed soil erosion at a European scale using different models, including the USLE equation. The average erosion reported for the entire Spanish territory was 3.79 tonnes per hectare and year, while in Galicia, these values were even lower. In the cultivation area, erosion was found to be below 1 tonne per hectare and year, supporting the findings of this study. According to the methodology of Lago-Olveira et al. (2025), midpoint characterisations can be obtained from the map values and subsequently converted into endpoint indicators to achieve a monetary evaluation of soil erosion. For example, for the plot, the occupation characterisation factor (CF_occ_) would be 0.12 tonnes per hectare and year, assuming pasture as the reference state, while the transformation characterisation factor (CF_trans_) would be 5.82 tonnes per hectare. Subsequently, the impact of occupation (I_occ_) and transformation (I_trans_), considering that land occupation for this crop lasts 20 years and the plot covers 2 hectares, would be 4.8 and 11.64 tonnes of soil, respectively. Finally, assuming a soil erosion control cost of €50.96 per tonne of soil (Lago-Olveira et al. 2025), the total costs generated by erosion due to cultivation would amount to €837.8, which translates to less than €42 per year. Assuming a cost per kilogram of dried hop cones of €4.89/kg (Ministry of Agriculture, Fisheries and Food 2021) and an average annual production of 1.98 tonnes per year, the cost of erosion control would represent less than 0.5% of the generated revenue.

Therefore, although the simulation indicates that erosion in the study area is relatively low and provides an economic perspective on this factor, it is based on maps and estimations from the literature, serving only as indicative data. For the development of effective erosion control strategies in the region, more detailed studies with real measurements should be conducted.

## Study limitations

Some limitations can be found in the models followed in this manuscript. The SDR model is based on the USLE equation (Renard et al. 1997), which has a limited scope as it only represents terrestrial erosion processes. Furthermore, the model is very sensitive to the k_b_ and IC_0_ parameters, which are not physically based. Additionally, due to the small number of parameters governing the equation, the results are highly sensitive, meaning that errors in the input parameters can significantly affect the displayed outcomes (Natural Capital Project 2024b).

On the other hand, the pollination model focuses exclusively on bees as pollinators, without including other significant groups such as wasps, butterflies or beetles (Natural Capital Project 2024a). Regarding the references used, the lack of a field observer at the plot who could provide real data led to the use of more general bibliographic sources, at the national (Spain) or even global level, to obtain information on the types of pollinators present, their abundance and their availability of nesting or foraging sites in the field.

Finally, the biodiversity loss estimation uses the model developed by Chaudhary et al. (2015), which relies on data from only five taxa (mammals, amphibians, reptiles, birds and plants), with other important groups with greater biodiversity, such as insects, not considered. Moreover, other important drivers of biodiversity loss, such as temperature rises and invasive species, are not considered in this model.

Consequently, while these models help provide a current estimate of these effects, which can serve as a starting point for establishing new practices focused on conservation and maintenance, these topics must be addressed in much greater depth to obtain conclusive results.

## Conclusions

This manuscript calculates the environmental profile of a hops cultivation system in the region of Galicia, Spain. The key environmental hotspots identified include the irrigation system, encompassing both its construction and the water, diesel and electricity required for its operation, as well as the production of fertilisers and the associated field emissions. To a lesser extent, field operations also contribute to the overall environmental impact. On the other hand, the abundance of pollinators is relatively high, suggesting that pest control strategies based on the use of these insects could be proposed, although pests (aphids, spider mite, etc.) in the region are not relevant at present. More sustainable practices in relation to pest and disease control could help to increase the viability of hop farm and preserve the environment. In terms of biodiversity loss, the impact is not particularly high compared to other agricultural studies, but nature-based solutions could be implemented to mitigate the habitat transformation impacts generated. Finally, the soil erosion observed in the hop farm is very low, being almost 88% lower than the national average, with an estimated cost for controlling this factor over the crop's lifespan of approximately €840.

Therefore, future studies should focus on finding sustainable production practices by addressing three key pillars: i) testing alternative water resources (reclaimed water or rainharvest systems) and pumps for irrigation, as the high energy and diesel consumption of the current ones has severe impacts (European Commission 2025); ii) studying the reduction of agrochemical use and, consequently, their emissions; and iii) reducing the number of machine operations and their diesel combustion impacts, as much as possible, through more natural strategies such as cover cropping, reduced tillage practices with diesel machinery or using autonomous robots. Moreover, the impact of these agricultural activities extends beyond the water circularity aspects, as advocated by the EU, over the environmental pillar, contributing to biodiversity loss and affecting ecosystem services such as those analysed in this study, ultimately leading to an overall improvement in the surrounding ecosystem.

## Supplementary Information

Below is the link to the electronic supplementary material.

ESM 1 (DOCX 99.8 KB)

## Data Availability

Data will be made available upon request.

## Abbreviations

NW: Northwest
ES: Ecosystem Services
LCA: Life Cycle Assessment
iLUC: Indirect Land Use Change
dLUC: Direct Land Use Change
GW: Global Warming
SOD: Stratospheric Ozone Depletion
TA: Terrestrial Acidification
FE: Freshwater Eutrophication
ME: Marine Eutrophication
TET: Terrestrial Ecotoxicity
FET: Freshwater Ecotoxicity
MET: Marine Ecotoxicity
WS: Water Scarcity
PSLglo: Potential Species Loss
