# Supplementary material for: Assessing environmental impacts and ecosystem services of Hops crop in Galicia, NW Spain: critical contributors for sustainable cultivation strategies
Source: Environ Sci Pollut Res Int. 2026 Mar 17;33(11):5133–47. doi: 10.1007/s11356-026-37623-0 (PMC13056744; doi:10.1007/s11356-026-37623-0)
Supplement: Supplementary file 1 — (DOCX 99.8 KB) [file 11356_2026_37623_MOESM1_ESM.docx]

**SUPPLEMENTARY MATERIAL**

**Assessing environmental impacts and ecosystem services of Hops crop in Galicia, NW Spain: critical contributors for sustainable cultivation strategies**

Adrián Agraso-Otero, Javier J. Cancela, María Fandiño, Ricardo Rebolledo-Leiva and Sara González-García

1. Field operations and agrochemicals application

The questionnaire filled out by the person responsible for the pilot, regarding field operations and agrochemical applications, is provided in Tables S1-S3.

**Table S1**. Field operations for the first year of hops cultivation.

| **Order** | **OPERATION** | | **TRACTOR** | **IMPLEMENTS** | | | Fuel consumption  (kg/ha∙operation) |
| --- | --- | --- | --- | --- | --- | --- | --- |
|  | Name | Time  (year) | Weight & Power  (kg) (kW) | Name | Weight  (kg) (products weights) | Operation rate  (h/ha) (1/CO) |  |
| 1 | Harrowing | 1 | 3740 kg  63,3 kw | Rotary harrow | 244 | 1.5 | 17.63 |
| 2 | Chemical fertilisation | 1 | 3740 kg  63,3 kw | Spraying machine | 580 | 2.0 | 23.50 |
| 3 | Ploughing | 1 | 3740 kg  63,3 kw | Plough | 940 | 2.0 | 23.50 |
| 4 | Smoothing | 1 | 3740 kg  63,3 kw | Rotary cultivator | 252 | 1.5 | 17.63 |
| 5 | Irrigation | 72 days (Jun-Aug) | 7,5 Kva | Irrigation Pump | - | - | 6.8 (x72) |
| 6 | Ridging | 1 | 3740 kg  63,3 kw | Side Ridging | 200 | 2.0 | 23.50 |
| 7 | Pest control | 3 times per year, once per month from March to May | 3740 kg  63,3 kw | Spraying machine | 700 | 1.0 | 11.75 (x3) |
| 8 | Pest control | 3 times per year, once per month from Jun to Aug | 3740 kg  63,3 kw | Spraying machine | 700 | 2.0 | 23.50 (x3) |
| 9 | Mechanical weed control | 3 times per year, once per month from Jun to Aug | 3740 kg  63,3 kw | Agricultural Brush Cutter with chains | 480 | 2.5 | 29.38 (x3) |

| **Table S2**. Field operations for the second and next years of hops cultivation. | | | | | | | |
| --- | --- | --- | --- | --- | --- | --- | --- |
| **Order** | **OPERATION** | | **TRACTOR** | **IMPLEMENTS** | | | Fuel consumption  (kg/ha∙operation) |
|  | Name | Time  (year) | Weight & Power  (kg) (kW) | Name | Weight  (kg) (Products weights) | Operation rate  (h/ha) (1/CO) |  |
| 1.1 | Chemical fertilisation | 1 | 3740 kg  63,3 kw | Spraying machine | 595 | 2.0 | 23.50 |
| 1.2 | Nitrogen fertilisation | 2 | 3740 kg  63,3 kw | Spraying machine | 295 | 1.5 | 17.63 (x2) |
| 2.1 | Herbicide treatment | 1 (Mar) Ridge | 3740 kg  63,3 kw | Spraying machine | 750 | 1.5 | 17.63 |
| 2.2 | Herbicide treatment | 1 (Aug) Chemical leaf removal | 3740 kg  63,3 kw | Spraying machine | 750 | 1.5 | 17.63 |
| 3 | Mechanical weed control | 6 times per year, once per month from March to Aug | 3740 kg  63,3 kw | Rotary cultivator | 252 | 2.0 | 23.50 (x6) |
| 4 | Ridging(‘Aporcado’) | 4-5 times | 3740 kg  63,3 kw | Side Ridging Ploughshares | 200 | 2.0 | 23.50 (x4-5) |
| 5.1 | Pest control | 10 times, every fifteen days from March to Sep (Mildiu) | 3740 kg  63,3 kw | Spraying machine | 950 | 1.0 | 11.75 (x10) |
| 5.2 | Pest control | 3 times, from Jul to Sep (Oidio) | 3740 kg  63,3 kw | Spraying machine | 1050 | 2.0 | 23.50 (x3) |
| 6 | Undo-Ridging  (‘Desaporcado’) | 1 | 3740 kg  63,3 kw | Side Ridging Ploughshares | 200 | 2.0 | 23.50 |
| 7 | Irrigation | 72 days (Jun-Aug) | 7,5 Kva | Irrigation Pump | - | - | 6.80 (x72) |
| 8 | Harvesting | 1 | 3740 kg  63,3 kw | Suspended cutter + Double axle agricultural trailers | 2580 | 28 | 329.00 |
| 9 | Cover crop (inter-row) | 1 | 3740 kg  63,3 kw | Trailed seeder | 450 | 2 | 23.50 |

**Table S3.** Agrochemicals used for hops cultivation.

| Type | Composition | Amount (kg/ha) | Number of treatments | Water per treatment (L) |
| --- | --- | --- | --- | --- |
| Fertilisers | | | | |
| NPK 8-15-15 |  | 900 | 1 | 0 |
| NAC27 |  | 250 | 1 | 0 |
| Ammonium nitrate 34,5 % |  | 200 | 1 | 1500 |
| Phytosanitary | | | | |
| Revus® | 250 g/l (23,40% p/p) Mandipropamid | 1 | 2 | 600 |
| Luna Sensation® | Fluopiram and Trifloxystrobin (250+250 g/L) | 0.6 | 1 | 600 |
| Caldo bordelés | Copper 20% | 4.5 | 1 | 600 |
| Sulphur | Sulphur 80% [WG] p/p | 5 | 1 | 600 |

1. SDR Model

All the data required for the SDR model in InVEST®, along with their sources, can be found in Table S4.

**Table S4.** Source of the data used for environmental analysis.

| Data | Format | Source |
| --- | --- | --- |
| Land use/Land cover | Raster | (European Environment Agency, 2018) |
| Biophysical table | CSV | (Marques et al., 2021; Panagos et al., 2015) |
| Digital elevation model | Raster | (European Space Agency, 2024) |
| Watershed | Vector | (Ministry for the Ecological Transition and the Demographic Challenge, 2025) |
| Rainfall erosivity | Raster | (Panagos et al., 2017, 2022) |
| Soil erodibility | Raster | (Panagos et al., 2014, 2022) |
| Threshold Flow Accumulation | Value (300) | (Lago-Olveira et al., 2025) |
| Borselli K Parameter | Value (2) | (Natural Capital Project, 2024) |
| Borselli IC0 Parameter | Value (0.5) | (Natural Capital Project, 2024) |
| Maximum SDR Value | Value (0.8) | (Natural Capital Project, 2024) |
| Maximum L Value | Value (122) | (Natural Capital Project, 2024) |

1. Life Cycle Aseesment

This section provides a table detailing the source of information used for the Life Cycle Assessment methodology (Table S5).

**Table S5.** Source of the data used for environmental analysis.

| Data | Source |
| --- | --- |
| Use of tractor and tillage | Farmers questionnaire |
| Amount of diesel | Farmers questionnaire |
| Amount and materials of the infrastructure | Farmers questionnaire |
| Amount and type of agrochemicals | Farmers questionnaire |
| Amount of water | Farmers questionnaire |
| Amount of pruning residues | Farmers questionnaire |
| Pump energy consumption | Farmers questionnaire |
| Yield | Farmers questionnaire |
| Agrochemicals emissions | Estimated (EMEP/EEA, 2023; European Commission, 2018; Faist Emmenegger et al., 2009; Intergovernmental Panel on Climate Change (IPCC), 2019; Prasuhn, 2006) |
| dLUC/iLUC emissions | Estimated (Schmidt et al., 2015) |
| Production of the tractor and tillage | Ecoinvent® database (Ecoinvent, 2024) |
| Diesel production and combustion emissions | Ecoinvent ® database (Ecoinvent, 2024) |
| Origin from water | Ecoinvent® database (Ecoinvent, 2024) |
| Production of electricity | Ecoinvent® database (Ecoinvent, 2024) |
| Production of the irrigation system | Ecoinvent® database (Ecoinvent, 2024) |
| Production of agrochemicals and infraestructure | Ecoinvent® database (Ecoinvent, 2024) |

Moreover, Table S6 lists the names of the processes in the Ecoinvent® database considered in the study.

**Table S6.** Ecoinvent® database processes considered in the background system.

| Activity | Process name |
| --- | --- |
| Tractor production | Tractor, 4-wheel, agricultural {GLO}\| market for \| Cut-off, U |
| Tillage production | Agricultural machinery, tillage {GLO}\| market for \| Cut-off, U |
| Diesel production | Diesel {RER}\| market group for \| Cut-off, U |
| Raw water | Water, unspecified natural origin, ES |
| Steel production  Fertilisers production | Steel, chromium steel 18/8 {GLO}\| market for \| Cut-off, U |
|  | Organic nitrogen fertiliser, as N {GLO}\| market for organic nitrogen fertiliser, as N \| Cut-off, U |
|  | Organic phosphorus fertiliser, as P2O5 {GLO}\| market for organic phosphorus fertiliser, as P2O5 \| Cut-off, U |
|  | Organic potassium fertiliser, as K2O {GLO}\| market for organic potassium fertiliser, as K2O \| Cut-off, U |
|  | Ammonium nitrate {RER}\| market for ammonium nitrate \| Cut-off, U  Calcium ammonium nitrate {RER}\| market for calcium ammonium nitrate \| Cut-off, U |
| Phytosanitaries production | Pesticide, unspecified {GLO}\| market for \| Cut-off, U |
| Infrastructure production | Steel, chromium steel 18/8 {GLO}\| market for \| Cut-off, U  Sawnwood, softwood, dried (u=10%), planed {RER}\| market for \| Cut-off, U  Polypropylene, granulate {RER}\| production \| Cut-off, U |

1. Indirect Land Use Change Calculation

This section provides a table detailing the step followed for the calculation of indirect land use changes (iLUC) (Table S7).

**Table S7.** Land use change calculations.

| iLUC | | | |
| --- | --- | --- | --- |
| **Step 1: land requirement** | Land requirement | 1 | ha/yr |
| **Steps 2-3: the potential net primary production** | NPP0 (Galicia) | 7 | t C/(ha·yr) |
|  | Potential use of the occupied land | market for arable land | |
| **Step 4-5: the productivity factor was obtained by dividing the NPP0 by the global average productivity** | Global average productivity | 6.11 | t C/(ha·yr) |
|  | Productivity factor | 1.15 | pw ha·yr/(ha·yr) |
| **Step 6: the current occupied area**  **(ha·yr) is converted into units of productivity weighted hectare years (pw**  **ha·yr), that is, 3.44 pw ha·yr per rotation** | Productivity weighted hectare years | 1.15 | *pw ha·yr* |
| **Step 7: the greenhouse gas (GHG) emissions** | iLUC emissions (mejor poner CF) | 0.042 | t CO_2_/ pw (ha·yr) |
|  | GHG | 0.04811784 | t CO_2_/ha |
|  | GHG | 48 | kg CO_2_/ha |

1. Uncertainty Assessment

This section presents a table summarizing the results of the uncertainty analysis performed on the inventory data used (Table S8).

**Table S8.** Results obtained from the Montecarlo method.

| Impact category | Unit | Mean | Median | Standard deviation |
| --- | --- | --- | --- | --- |
| GW | kg CO_2_ eq | 2.96 | 2.94 | 0.2 |
| SOD | mg CFC_11_ eq | 29.8 | 29.8 | 0.58 |
| TA | g SO_2_ eq | 34.9 | 34.8 | 0.79 |
| FE | g P eq | 1.09 | 1.04 | 0.28 |
| ME | g N eq | 15.7 | 15.7 | 0.01 |
| TET | kg 1,4-DCB | 14.6 | 14.2 | 2.79 |
| FET | kg 1,4-DCB | 0.14 | 0.13 | 0.06 |
| MET | kg 1,4-DCB | 0.18 | 0.17 | 0.08 |

References

Ecoinvent. (2024). *Data with purpose*. https://ecoinvent.org/

EMEP/EEA. (2023). *EMEP/EEA air pollutant emission inventory guidebook 2023. Technical guidance to prepare national emission inventories*.

European Commission. (2018). *Product Environmental Footprint Category Rules Guidance. PEFCR Guid. Doc*.

European Environment Agency. (2018). *CORINE Land Cover 2018*. Copernicus Land Monitoring Service. https://land.copernicus.eu/pan-european/corine-land-cover/clc2018

European Space Agency. (2024). *Copernicus Global Digital Elevation Model*. OpenTopography. https://doi.org/10.5069/G9028PQB

Faist Emmenegger, M., Zah, R., & Reinhard, J. (2009). *Sustainable Quick Check for Biofuels (SQCB): A Web-based tool for streamlined biofuels’ LCA*. 297–303.

Intergovernmental Panel on Climate Change (IPCC). (2019). *N2O Emissions From Managed Soils, and Co2 Emissions From Lime and Urea Application, 2019 Refinement to the 2006 IPCC Guidelines for National Greenhouse Gas Inventories*.

Lago-Olveira, S., Moreira, M. T., & González-García, S. (2025). Quantifying spatially explicit LCA midpoint characterization factors to assess the impact of specific farming practices on ecosystem services. *Ecosystem Services*, *71*, 101686. https://doi.org/10.1016/J.ECOSER.2024.101686

Marques, S. M., Campos, F. S., David, J., & Cabral, P. (2021). Modelling sediment retention services and soil erosion changes in Portugal: A spatio-temporal approach. *ISPRS International Journal of Geo-Information*, *10*(4). https://doi.org/10.3390/ijgi10040262

Ministry for the Ecological Transition and the Demographic Challenge. (2025). *Mapa de cuencas de los ríos atlánticos del norte*. Spanish Government. https://www.mapama.gob.es/app/descargas/descargafichero.aspx?f=A_cuencas_rios_Atl_Norte.zip

Natural Capital Project. (2024). *InVEST Sediment Delivery Ratio (SDR) model*. http://releases.naturalcapitalproject.org/invest-userguide/latest/en/sdr.html#data-needs

Panagos, P., Borrelli, P., Meusburger, K., van der Zanden, E. H., Poesen, J., & Alewell, C. (2015). Modelling the effect of support practices (P-factor) on the reduction of soil erosion by water at European scale. *Environmental Science and Policy*, *51*, 23–34. https://doi.org/10.1016/j.envsci.2015.03.012

Panagos, P., Borrelli, P., Meusburger, K., Yu, B., Klik, A., Lim, K. J., Yang, J. E., Ni, J., Miao, C., Chattopadhyay, N., Sadeghi, S. H., Hazbavi, Z., Zabihi, M., Larionov, G. A., Krasnov, S. F., Gorobets, A. V., Levi, Y., Erpul, G., Birkel, C., … Ballabio, C. (2017). Global rainfall erosivity assessment based on high-temporal resolution rainfall records. *Scientific Reports*, *7*(1). https://doi.org/10.1038/s41598-017-04282-8

Panagos, P., Meusburger, K., Ballabio, C., Borrelli, P., & Alewell, C. (2014). Soil erodibility in Europe: A high-resolution dataset based on LUCAS. *Science of the Total Environment*, *479–480*(1), 189–200. https://doi.org/10.1016/j.scitotenv.2014.02.010

Panagos, P., Van Liedekerke, M., Borrelli, P., Köninger, J., Ballabio, C., Orgiazzi, A., Lugato, E., Liakos, L., Hervas, J., Jones, A., & Montanarella, L. (2022). European Soil Data Centre 2.0: Soil data and knowledge in support of the EU policies. *European Journal of Soil Science*, *73*(6). https://doi.org/10.1111/ejss.13315

Prasuhn, V. (2006). *Erfassung der PO4-Austräge für die Ökobilanzierung - SALCA-Phosphor. Agroescope Reckenholz 20.*

Schmidt, J. H., Weidema, B. P., & Brandão, M. (2015). A framework for modelling indirect land use changes in Life Cycle Assessment. *Journal of Cleaner Production*, *99*, 230–238. https://doi.org/10.1016/J.JCLEPRO.2015.03.013
